# Purification and Characterization of Hemagglutinating Proteins from Poker-Chip Venus (*Meretrix lusoria*) and Corbicula Clam (*Corbicula fluminea*)

**DOI:** 10.1100/2012/906737

**Published:** 2012-05-01

**Authors:** Chin-Fu Cheng, Shao-Wen Hung, Yung-Chung Chang, Ming-Hui Chen, Chen-Hsuan Chang, Li-Tse Tsou, Ching-Yu Tu, Yu-Hsing Lin, Pan-Chen Liu, Shiun-Long Lin, Way-Shyan Wang

**Affiliations:** ^1^Department of Veterinary Medicine, College of Veterinary Medicine, National Chung Hsing University, Taichung 402, Taiwan; ^2^Agricultural Biotechnology Research Center, Academia Sinica, Taipei 115, Taiwan; ^3^Agricultural Chemicals and Toxic Substances Research Institute, Council of Agriculture, Executive Yuan, Taichung 413, Taiwan; ^4^Nursing Department, Yuanpei University, Hsinchu 300, Taiwan

## Abstract

Hemagglutinating proteins (HAPs) were purified from Poker-chip Venus (*Meretrix lusoria*) and Corbicula clam (*Corbicula fluminea*) using gel-filtration chromatography on a Sephacryl S-300 column. The molecular weights of the HAPs obtained from Poker-chip Venus and Corbicula clam were 358 kDa and 380 kDa, respectively. Purified HAP from Poker-chip Venus yielded two subunits with molecular weights of 26 kDa and 29 kDa. However, only one HAP subunit was purified from Corbicula clam, and its molecular weight was 32 kDa. The two Poker-chip Venus HAPs possessed hemagglutinating ability (HAA) for erythrocytes of some vertebrate animal species, especially tilapia. Moreover, HAA of the HAP purified from Poker-chip Venus was higher than that of the HAP of Corbicula clam. Furthermore, Poker-chip Venus HAPs possessed better HAA at a pH higher than 7.0. When the temperature was at 4°C–10°C or the salinity was less than 0.5‰, the two Poker-chip Venus HAPs possessed better HAA compared with that of Corbicula clam.

## 1. Introduction

Along the coastline of west Taiwan and southeast China, Poker-chip Venus (*Meretrix lusoria*) has become one of the most important commercial marine bivalves [1]. Furthermore, the freshwater bivalve Corbicula clam (*Corbicula fluminea*) can be found in several rivers in Taiwan. Bivalves (Poker-chip Venus and Corbicula clam) are cheap and highly nutritious, making them one of the most popular sources of daily protein in Taiwan and China [1]. Bivalves also act as environmental indicators in monitoring ocean contamination [2, 3]. Mass mortalities of Poker-chip Venus with unknown cause in hard clam fisheries have been reported [4]. In Taiwan, outbreaks typically occur during seasonal changes resulting in heavy economic loss [2].

Some lectins from invertebrates were reported to be involved in various biological responses, for instance, promotion of antibacterial activity [5–7], phagocytosis [8–10], nodule formation [11], and activation of the proPO system [12–14]. Lectins are proteins that have the ability to recognize and bind to specific carbohydrate chains on the target cells, which is then followed by the formation of ion-permeable pores in the cell membrane through oligomerization of the protein. After the formation of the pores, erythrocytes are ruptured by colloid osmotic shock. Furthermore, they are present in almost all living organisms, and a lot of lectins have been found in tissues and body fluids in various animals [15].

Invertebrates lack adaptive immune systems, but they have developed various defense systems of innate immunity that recognize antigens on the surface of potential pathogens, including a set of humoral and cellular immune reactions [1]. Innate immunity, not only in vertebrates but also in invertebrates, is now attracting considerable attention. Recognition of nonself materials in the innate immune system is mediated by a group of serum proteins, which recognize and bind to different molecules on the surface of pathogens and trigger a series of immune responses, leading to the activation of the host-defense system [2, 3]. Because there are few data on hemagglutinating proteins in bivalves, the aim of this study was to purify and characterize HAPs from Poker-chip Venus and Corbicula clam.

## 2. Materials and Methods

### 2.1. Preparation of Bivalves

Approximately 1,000 live adult Poker-chip Venus clams, *Meterix lusoria*, and 1,000 Corbicula clams, *Corbicula fluminea*, were purchased from an aquaculture farm in Taiwan. The average shell length and the mean wet weight of Corbicula clams were 27.5 ± 0.1 mm and 1.2 ± 0.01 g (mean ± standard error), respectively. The average shell length and the mean wet weight of Poker-chip Venus clams were 45.0 ± 0.1 mm and 16.0 ± 0.01 g, respectively. Poker-chip Venus clams were cultured in 15‰–18‰  seawater (volume: 6,000 L) over a 10 cm sea sand layer and Corbicula clams were cultured in fresh water (volume: 6,000 L) over a 10 cm fresh water sand layer. They were fed twice daily with fresh feed (Marine Snow plankton diet, Two Little Fishies, USA; 1% of the body weight), prior to beginning the study.

### 2.2. Serum Collection

Hemolymph was taken from the posterior adductor muscles of Poker-chip Venus and Corbicula clams with a 3 mL sterile syringe (26-gauge needle) and centrifuged at 2,000 ×g for 30 min at 4°C to separate the clot. Serum samples were then stored at −80°C until use.

### 2.3. Serum Condensation

Collected serum samples were put into the dialysis membrane (12–14 kDa; Spectrum Medical Industries, Los Angeles, CA, USA) and dialyzed with pH 7.2 PBS at 4°C for two days to remove salt. Dialyzed serums were then condensed with ultrafiltration membrane (Centriprep-100; Amico, Beverly, MA, USA) at 800 ×g, 4°C for 3 h. After performing condensation ten times, serum samples were stored at −80°C until use.

### 2.4. The Fast Protein Liquid Chromatography System

Condensed serums were added into the C16/70 column (1.6 × 57.5 cm; Pharmacia Biotech., Sollentuna, Sweden) with Sephacryl S-300 (Pharmacia) linked to the fast protein liquid chromatography system (FPLC system; Pharmacia). Before starting the FPLC system, serums were first filtrated through the 0.45 *μ*m membrane, then 0.5 mL filtrated and condensed serums were placed into the Sephacryl S-300. The running buffer was PBS, which was composed of 1.34 g/L KH_2_PO_4_, 7.16 g/L Na_2_HPO_4_, 8.76 g/L NaCl, and 0.2 g/L NaN_3_, was used to separate serum proteins from serums. The flow rate was 0.8 mL/min and the samples were analyzed using an ultraviolet detector at a wavelength of 280 nm. After evaluating HAA of samples, HAA-positive serums were collected and stored at −80°C until use.

### 2.5. Molecular Weight Measurement

HMW gel filtration calibration kit (Pharmacia) was used to determine the molecular weight of the serum proteins. Kit included standard samples (molecular weights) such as blue dextran (2,000 kDa), thyroglobulin (669 kDa), ferritin (440 kDa), catalase (232 kDa), and aldolase (158 kDa). A volume of 0.5 mL standard molecule weight samples (30 *μ*g/mL) and condensed proteins were added into the FPLC system, and their flow volume was calibrated. *V*_*o*_ was used to indicate flow volume of blue dextran, and *V*_*e*_ represented the flow volumes of other standard molecule weight samples. The total volume of Sephacryl S-300 in C16/70 column was termed *V*_*t*_. Finally, the formula, *K*_av_ was calculated (*K*_av_ = (*V*_*e*_ − *V*_*o*_)/(*V*_*t*_ − *V*_*o*_)). According to *K*_av_ of standard molecular weight samples and the log values of their molecular weights, standard curves of standard molecule weight samples in the FPLC system could be calculated. According to the standard curve of standard molecular weight samples, the molecular weight of the condensed proteins could be calculated.

### 2.6. Sodium Dodecyl Sulfate-Polyacrylamide Gel Electrophoresis (SDS-PAGE) and Native PAGE

SDS-PAGE and native PAGE were used to classify the subunits of the HAA-positive HAPs. Samples (30 *μ*g/mL) were boiled for 5 min before loading them onto the 12% SDS-polyacrylamide gel and native polyacrylamide gel. After electrophoresis for 3 h at 80 V, the gel was fixed with fixing buffer (30% methanol and 5% acetic acid) for 10 min then washed with double-distilled water three times. The gel was stained with silver staining kit (Bio-Rad, Hercules, CA, USA), and the reaction was stopped with 10% acetic acid. Finally, the molecular weights of HAPs were calculated according to the standard curves of the standard molecular weight samples.

### 2.7. Protein Concentration Measurement

The concentrations of the HAPs were measured using a protein assay reagent kit (Bio-Rad). Bovine serum albumin (BSA; Bio-Rad) was used as the standard sample. To detect the optical density of BSA, spectrophotometer (Beckmen, CA, USA) was used at a wavelength of 595 nm and then the regression curve of BSA was calculated. Finally, 20 *μ*L HAPs was reacted with 1 mL protein assay reagent and the concentration of the HAPs, on the basis of the regression curve of BSA, were calculated.

### 2.8. Hemagglutinating Activity (HAA)

Using a modified version of the method described by Odo et al. [16] erythrocytes were isolated from eight different animal species, including human, horse, sheep, canine, rabbit, chicken, tilapia, and grass carp. Blood was anticoagulated with 1,000 IU heparin (Sigma), centrifuged at 160 ×g for 10 min, and then, the supernatant was discarded. Later, cell concentration of 4 × 10^7^ erythrocytes per mL was prepared by a 3% (v/v) suspension of a variety of animal erythrocytes which were prepared in Tris-buffered saline with calcium (TBS-Ca) (50 mM Tris-HCl, pH 7.5, 100 mM NaCl, and 10 mM CaCl_2_). After 2-fold serial dilutions in the same buffer, the sample (50 *μ*L) was mixed with 50 *μ*L of the cell suspension in 96-well U-bottom microtiter plates (Corning), and hemagglutination was observed after incubation for 1 h at 25°C under a light microscope (Olympus). The endpoint was defined as the highest dilution showing complete hemagglutination. The titer as a percentage of the control titer was calculated by dividing the titer of the control by the titer of the test and multiplying by 100. Furthermore, we also assessed the HAA of HAPs at different temperatures (4°C, 10°C, 20°C, 30°C, 40°C, and 50°C), different pH values (pH 1.0–14.0), and different salinities (5‰, 10‰, 15‰, 20‰, 25‰, 30‰, 35‰, and 40‰) using the same method described above.

### 2.9. Inhibition of Vibrio anguillarum Cocultured with HAPs

Two hundred *μ*g/mL of purified proteins were cocultured with 1 mL *Vibrio anguillarum* (2 × 10^5^ CFU/mL) at 25°C for 0 h, 2 h, 3 h, 4 h, 5 h, and 6 h. Finally, a spectrophotometer was used to measure the optical density at a wavelength of 600 nm.

### 2.10. Neutralization of IPNV Cocultured with HAPs

Two-fold serial dilutions of HAPs (500 *μ*L, 200 *μ*g/mL) in PBS were mixed with 500 *μ*L of IPNV (2,000 TCID_50_/mL) in 96-well microtiter plates (Corning) at 20°C for 2 h. Then, 100 *μ*L of HAPs-IPNV cocultured medium was added into CHSE-214 (Chinook salmon embryo-214) cell line in 96-well microtiter plates (Corning) at 20°C for 18 h, 24 h, 36 h, and 48 h. Furthermore, only 1,000 TCID_50_/mL IPNV cultured medium and 100 *μ*g/mL HAPs were used as positive and negative groups, respectively. Finally, the cytopathic effect (CPE) was observed under a microscope (Olympus) and 50% neutralization dose (ND_50_) was also assessed.

### 2.11. Statistical Analysis

All assays were performed in triplicate. Statistical analysis system (SAS), Duncan's new multiple range test, Kruskal-Wallis one-way analysis of variance (ANOVA), and Student's *t*-test were performed to assess overall differences between the different treatments. A *P* value less than 0.05 was considered significant.

## 3. Results

### 3.1. Serum Biochemistry Values of Poker-Chip Venus and Corbicula Clam

Table 1 shows that the concentration of total protein in Corbicula clam hemolymph (0.16 ± 0.001 g/dL) was significantly higher than that in Poker-chip Venus (0.06 ± 0.008 g/dL) (*P* < 0.05). Furthermore, the concentrations of glucose, ions (Na^+^, K^+^, Cl^−^, Mg^2+^, and inorganic phosphorous) in Poker-chip Venus were significantly higher than those in Corbicula clam (*P* < 0.05). However, the concentrations of Ca^2+^ ion in Poker-chip Venus and Corbicula clam hemolymph were not significantly different.

### 3.2. Purification and Identification of Molecular Weight of HAPs Purified from Poker-Chip Venus and Corbicula Clam

**Figure 1:**
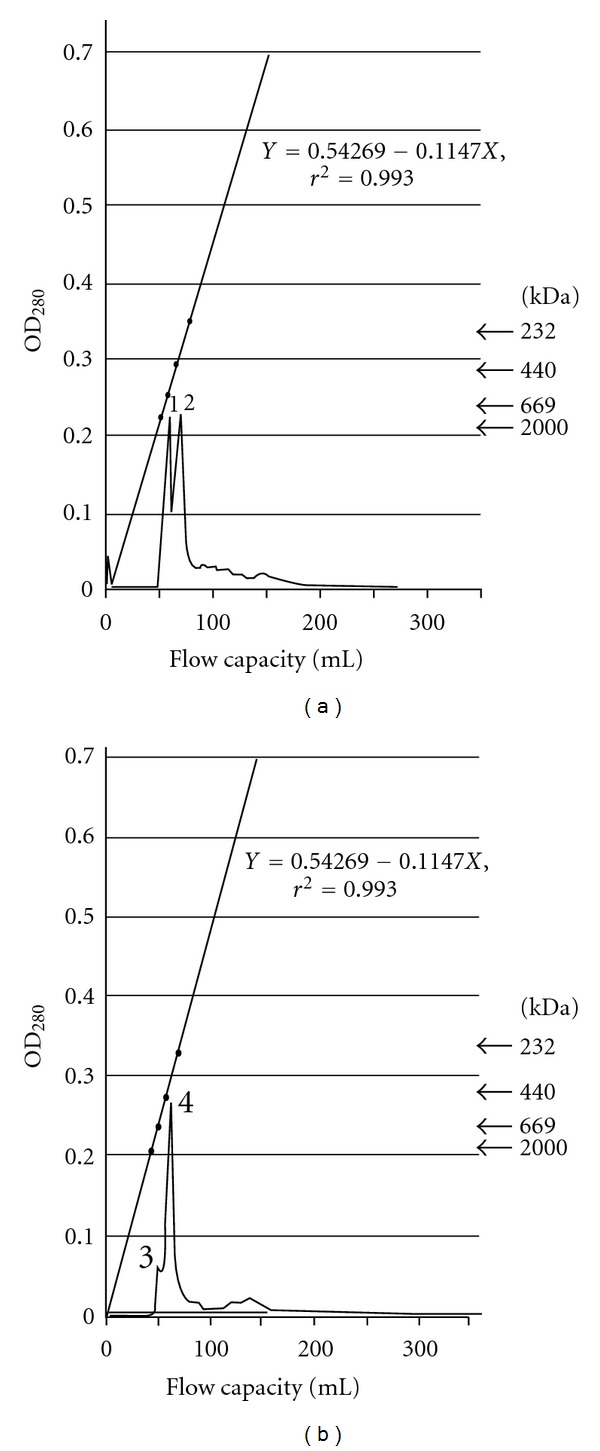
Gel filtration chromatography of the hemolymph proteins from Poker-chip Venus (a) and Corbicula clam (b). The peaks 1, 2, 3, and 4 indicate different proteins in hemolymph.

**Figure 2:**
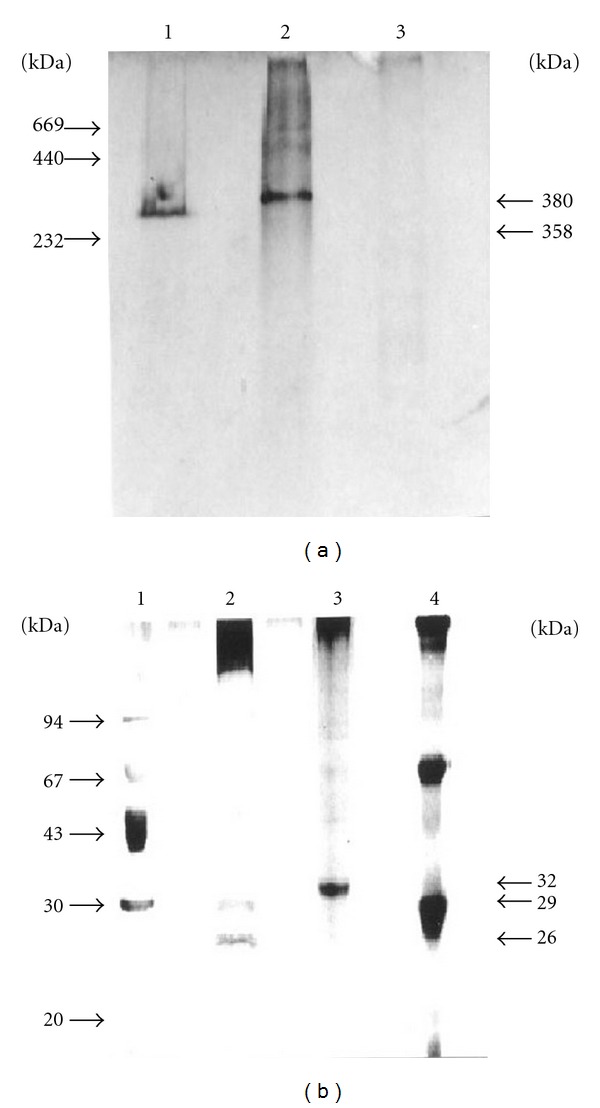
The native PAGE (a) and SDS-PAGE (b) of the hemagglutinating proteins purified from Poker-chip Venus and Corbicula clam. (a) Lane 1: the hemagglutinating protein purified from Poker-chip Venus; lane 2: the hemagglutinating protein purified from Corbicula clam; lane 3: bovine IgM. (b) Lane 1: LMW standard markers; lane 2: the hemagglutinating protein purified from Poker-chip Venus; lane 3: the hemagglutinating protein purified from Corbicula clam; lane 4: bovine IgM.

The results obtained by FPLC system showed two obvious peaks (Figure 1). We collected the second-peak sample and determined the molecular weights. Finally, we obtained molecular weights of 358 kDa and 380 kDa in Poker-chip Venus and Corbicula clam, respectively (Figure 1). The equations of regression curves for these molecular weights were also calculated, as shown in Figure 1. Furthermore, we also confirmed their molecular weights using native PAGE and found values of 358 kDa and 380 kDa for Poker-chip Venus and Corbicula clam, respectively (Figure 2(a)). Finally, we used SDS-PAGE to obtain the subunit molecular weights of HAPs from Poker-chip Venus, which were 26 kDa and 29 kDa, and in Corbicula clam the subunit molecular weight was 32 kDa (Figure 2(b)).

### 3.3. Bioactivities of HAPs Purified from Poker-Chip Venus and Corbicula Clam

**Figure 3:**
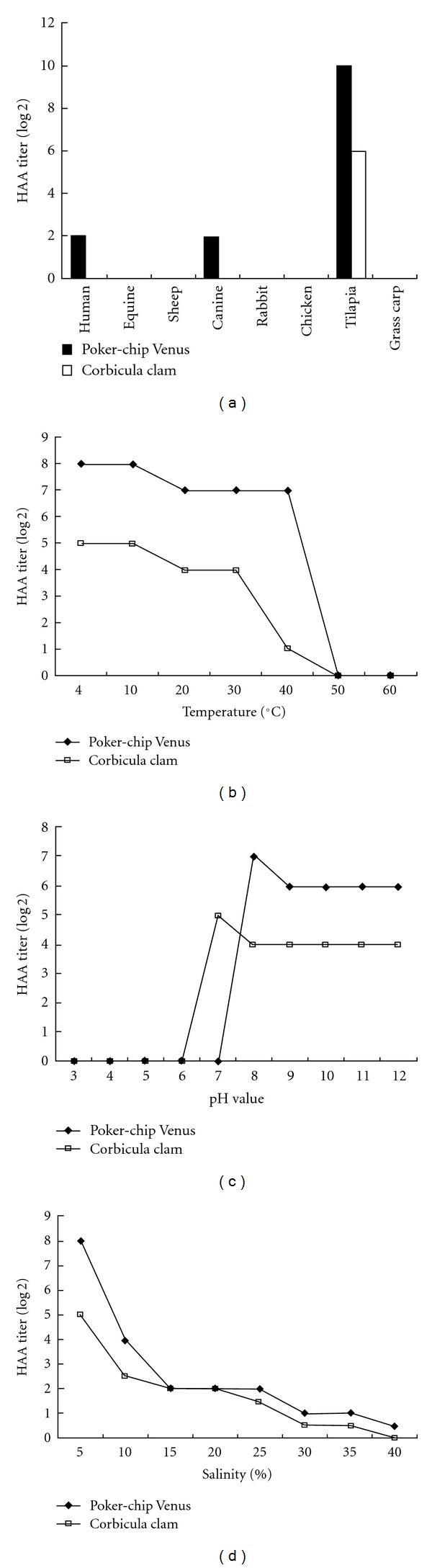
Effect of the hemagglutinating activity with the hemagglutinating proteins purified from Poker-chip Venus and Corbicula clam under several different conditions. (a) HAA titer of the hemagglutinating proteins in different animal species. (b) HAA titer of the hemagglutinating proteins at different temperatures. (c) HAA titer of the hemagglutinating proteins at different pH values. (d) HAA titer of the hemagglutinating proteins at different salinities.

Figure 3(a) shows that the purified Poker-chip Venus HAP could hemagglutinate tilapia, human, and canine erythrocytes, and tilapia erythrocytes showed the strongest hemagglutination. However, the purified Corbicula clam HAP could only hemagglutinate tilapia erythrocytes.

According to the results in Figure 3(b), the purified Poker-chip Venus and Corbicula clam HAPs possessed the highest HAA titer at less than 10°C. The purified Poker-chip Venus HAP possessed a higher HAA titer than that of the purified Corbicula clam HAP at less than 50°C. Furthermore, there was no HAA of the purified Poker-chip Venus and Corbicula clam HAPs when the temperature was above 50°C.

Figure 3(c) shows the purified Poker-chip Venus and Corbicula clam HAPs possessed the highest HAA titers at pH 7.0 and pH 8.0, respectively. The purified Poker-chip Venus HAP possessed a higher HAA titer than that of the purified Corbicula clam HAP at pH 8.0–12.0. Furthermore, there was no HAA of the purified Poker-chip Venus HAP at less than pH 7.0 or of the purified Corbicula clam HAP at less than pH 6.0.

It can be seen in Figure 3(d) that the purified Poker-chip Venus and Corbicula clam HAPs possessed the highest HAA titer at 5‰  salinity. When the salinity was increased, the HAA of the purified Poker-chip Venus and Corbicula clam HAPs was decreased. When the salinity reached 40‰, there was almost no HAA in the purified Poker-chip Venus and Corbicula clam HAPs.

### 3.4. The Purified Poker-Chip Venus and Corbicula Clam HAPs Affected Vibrio anguillarum Growth

**Figure 4:**
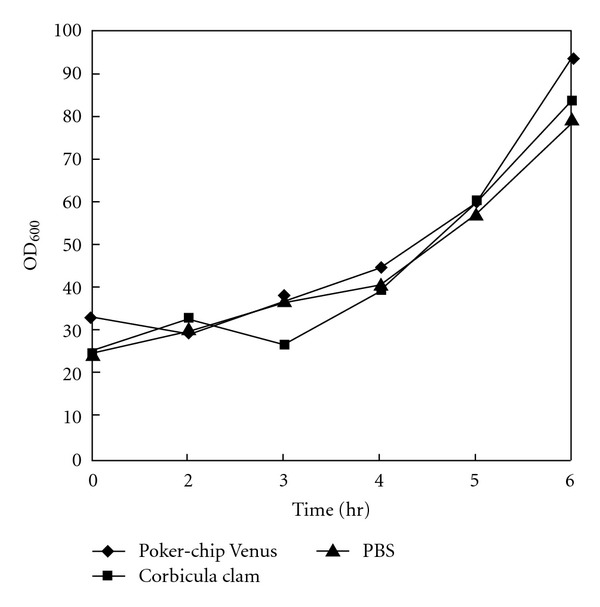
Effect of the growth of* Vibrio anguillarum* with the hemagglutinating proteins purified from Poker-chip Venus and Corbicula clam in several different culture time. PBS-treated group was used as control.

As shown in Figure 4, the purified Poker-chip Venus and Corbicula clam HAPs did not possess the ability to inhibit *Vibrio anguillarum *growth. In fact, these HAPs appeared to induce *Vibrio anguillarum *growth.

## 4. Discussion

Belogortseva et al. [17] reported that HAP could be purified using affinity chromatography on acid-treated Sepharose 6B followed by gel filtration on Sephacryl S-200. Odo et al. [16] purified HAP using affinity chromatography with Sepharose 4B followed by gel filtration. Tunkijjanukij et al. [18] reported that HAP could be purified using bovine submaxillary mucin conjugated to CNBr-activated Sepharose 4B then applying gradient-gel electrophoresis and gel filtration on Biogel and Superose. In the present study, HAPs were purified successfully at a high purity using affinity chromatography on Sephacryl S-300 as the affinity matrix, which was linked to the FPLC system. According to results of gel filtration, there were two peaks, but only the second-peak HAP possessed HAA.

HAPs comprising several subunits have been reported in many invertebrate species. Alpuche et al. [19] reported that HAP purified from the white shrimp, *Litopenaeus setiferus* hemolymph was a heterotetramer of two 80 kDa and two 52 kDa subunits. Murali et al. [20, 21] reported that a natural agglutinin purified from serum of the hermit crab, *Diogenes affinis* possessed four subunits (51, 49, 42, and 39 kDa). Tunkijjanukij et al. [18] reported that horse mussel HAP possessed three subunits of 14, 17.5, and 20 kDa. Moreover, Odo et al. [16] reported that HAP purified from the marine bivalve, *Tridacna derasa* (Roding) possessed two subunits of 23 and 46 kDa. In the present investigation, two subunits of 26 kDa and 29 kDa were found in Poker-chip Venus HAP and only one subunit of 32 kDa in Corbicula clam HAP.

Many studies have been conducted on purification of HAP from invertebrates, including protozoan parasite [22, 23], insect [24–26], urchin [27], shrimp [19, 28, 29], crab [30], and mollusk [31]. Few studies have investigated HAP in shellfish other than crustaceans, and HAP data on bivalves are particularly scarce. Suzuki and Mori [32] reported that hemolymph lectin of the pearl oyster, *Pinctada fucata* could hemagglutinate horse erythrocytes. Odo et al. [16] reported that HAP purified from the marine bivalve, *Tridacna derasa* (Roding), could agglutinate various animal species erythrocytes. In addition, Tunkijjanukij et al. [18] found that HAP purified from horse mussel (*Modiolus modiolus*) could agglutinate human and horse erythrocytes. Furthermore, Belogortseva et al. [17] demonstrated that HAP purified from sea mussel, *Crenomytilus grayanus*, could agglutinate all types of human erythrocytes together with those of mouse and rabbit. In the current study, our purified HAP from Poker-chip Venus showed serological activity against RBC of some animal species, including human, canine, and tilapia, and HAP purified from Corbicula clam only showed a response against tilapia RBC. According to the HAA of HAPs in Poker-chip Venus and Corbicula clam, the freshwater clam possessed a different HAA on animal species compared with that of the seawater clam.

Odo et al. [16] reported that HAP purified from the marine bivalve possessed a HAA which decreased dramatically below pH 6.5, but which reincreased to the original level when pH value was 7.0. Belogortseva et al. [17] reported that HAP purified from sea mussel possessed an HAA that was independent of Ca^2+^ and Mg^2+^ ions. Significantly increased HAA was observed between pH 8–10. In the present study, the purified HAP from Poker-chip Venus possessed the highest HAA at pH 8–12, and HAP from Corbicula clam possessed the highest HAA at pH 7–12. The highest HAAs of Poker-chip Venus and Corbicula clam HAP were observed at pH 8.0 and pH 7.0, respectively. Furthermore, we also found temperature and salinity affected HAA. Regarding the effects of temperature, significant HAA of Poker-chip Venus and Corbicula clam HAPs were observed between 4°C–40°C and 4°C–30°C, respectively. The highest HAA of Poker-chip Venus and Corbicula clam HAPs were both observed at 4°C–10°C. In addition, the highest HAA of Poker-chip Venus and Corbicula clam HAP were both observed at a salinity of 5‰. HAAs were decreased proportionally with increased salinity in Poker-chip Venus and Corbicula clam HAPs. Finally, our purified HAP did not possess anti-*V. anguillarum* and viral neutralization (for infectious pancreatic necrosis virus) activities (data not shown).

The results of this study may be of use as reference data on HAPs of Poker-chip Venus and Corbicula clam. Further studies are needed to understand the amino acid sequences and conjugation of HAP and their subunits.

## Figures and Tables

**Table 1:** The biochemical properties of Poker-chip Venus and Corbicula clam hemolymph.

	No.	Poker-chip Venus	Corbicula clam
Total protein (g/dL)	25	0.06 ± 0.008	0.16 ± 0.001*
Glucose (mg/dL)	25	8.1 ± 0.35*	3.6 ± 0.34
Na^+^ ion (mmol/L)	25	228.2 ± 0.50*	23.6 ± 0.38
K^+^ ion (mmol/L)	25	6.8 ± 0.10*	1.1 ± 0.04
Cl^−^ ion (mmol/L)	25	241.7 ± 0.81*	8.4 ± 0.25
Ca^2+^ ion (mg/dL)	25	20.5 ± 0.07	18.6 ± 0.85
Mg^2+^ ion (mg/dL)	25	7.5 ± 0.03*	5.3 ± 0.20
Inorganic phosphorus (mg/dL)	25	12.5 ± 0.82*	3.8 ± 0.27

*P < 0.05.
